# Nested Named Entity Recognition Based on Dual Stream Feature Complementation

**DOI:** 10.3390/e24101454

**Published:** 2022-10-12

**Authors:** Tao Liao, Rongmei Huang, Shunxiang Zhang, Songsong Duan, Yanjie Chen, Wenxiang Ma, Xinyuan Chen

**Affiliations:** College of Computer Science and Engineering, Anhui University of Science and Technology, Huainan 232001, China

**Keywords:** named entity recognition, nested structure, dual-flow feature complementary, neural network

## Abstract

Named entity recognition is a basic task in natural language processing, and there is a large number of nested structures in named entities. Nested named entities become the basis for solving many tasks in NLP. A nested named entity recognition model based on dual-flow features complementary is proposed for obtaining efficient feature information after text coding. Firstly, sentences are embedded at both the word level and the character level of the words, then sentence context information is obtained separately via the neural network Bi-LSTM; Afterward, two vectors perform low-level feature complementary to reinforce low-level semantic information; Sentence-local information is captured with the multi-head attention mechanism, then the feature vector is sent to the high-level feature complementary module to obtain deep semantic information; Finally, the entity word recognition module and the fine-grained division module are entered to obtain the internal entity. The experimental results show that the model has a great improvement in feature extraction compared to the classical model.

## 1. Introduction

Named Entity Recognition (NER) refers to identifying and judging a word with special meaning and its type from unstructured text, such as a person’s name, location, organization name, and proper noun [1,2,3]. NER is of great significance in the process of natural language processing (NLP), playing a crucial role in a wide range of downstream natural language processing tasks, including relation extraction [4,5], information retrieval [6], machine translation [7], aspect-based sentiment analysis [8,9], question answering system [10,11]. Previous research has focused on non-nested named entities (Flat NER). When dealing with non-nested named entities, the current method generally is to solve the problem as sequence annotation, but this method cannot accurately identify entities with nested structures. For example, in an English sentence such as “He used to study in University of Cambridge.”, where Cambridge is a location entity, embedded in another longer organization entity: University of Cambridge. The nature of phrasal representation in natural languages makes named entities usually nested, therefore, more and more people consider and study nested named entities.

In recent years, with the wide application of deep learning in NLP, many new approaches to nested NER have emerged. The hierarchical approach addresses this task through multi-level sequential labeling, that is, dividing entities into multiple levels, where the number of labeled layers indicates the depth of entity nesting. Shibuya and Hovy [12] adopted a hierarchical method, repeatedly detected the internal entities by applying the traditional conditional random field (CRF) [13], and then searched out the best path from the space to obtain the next layer of entities. Li and Feng [14] proposed the idea of taking the nested NER task as reading comprehension and achieved good results on GENIA and other nested entity recognition datasets. Another method of transformation converts nested named entities into linear structures, followed by sequence markup. For example, Muis and Lu [15] proposed a new method based on hypergraph, and Katiyar and Cardie [16] also based their work on the hypergraph method and used LSTM [17,18] for linear learning in a greedy way. It can be found that most of the current methods use the joint embedding of word-level representation and character-level representation to obtain feature information, which does not consider the dependence between word features and character features so that the hidden feature information cannot be obtained efficiently.

To solve the problem that the hidden information of word-level embedding feature vectors and character-level embedding feature vectors is not enough, a dual-stream feature complementation mechanism is proposed. The main contributions of this paper are as follows: (1) a novel dual-stream feature complementary nested named entity recognition method is proposed. Different from the existing methods, which directly integrate character embedding vectors and word embedding vectors during embedding, this paper puts the two embedding vectors into Bi-LSTM [19] to obtain text context features respectively and then uses a bidirectional feature complementation mechanism to explore the maximum information gain between the two feature vectors. (2) To implement the feature complementation mechanism proposed in this paper, a low-level bidirectional complementation module and a high-level bidirectional complementation module are designed to realize the bidirectional feature information complementation mechanism from two dimensions respectively. (3) Through a comparative analysis of the GENIA dataset, the conclusion obtained in this paper is that compared with other classical experiments, the experimental results in this paper are the best.

Overall, the main contributions of this proposed work are as follows:

- a novel dual-stream feature complementary nested named entity recognition method is proposed. Different from the existing methods, which directly integrate character embedding vectors and word embedding vectors during embedding, this paper puts the two embedding vectors into Bi-LSTM [19] to obtain text context features respectively and then uses a bidirectional feature complementation mechanism to explore the maximum information gain between the two feature vectors.
- To implement the feature complementation mechanism proposed in this paper, a low-level bidirectional complementation module and a high-level bidirectional complementation module are designed to realize the bidirectional feature information complementation mechanism from two dimensions respectively.
- Through a comparative analysis of the GENIA dataset, the conclusion obtained in this paper is that compared with other classical experiments, the experimental results in this paper are the best.

The remainder of this article is organized as follows. Section 2 reviews the related work, and the our model for NER is presented in Section 3. Comprehensive experimental results are discussed in Section 4 for performance evaluations compared with SOTA methods. Finally, concluding remarks and future directions are given in Section 5.

## 2. Related Work

### 2.1. Named Entities

Existing methods for identifying non-nested named entities usually treat the NER task as a sequence labeling problem. Compared with feature-based methods, deep learning is helpful in automatically discovering hidden features. Hammerton [20] uses LSTM for NER work for the first time. Collobert and Weston et al. [21] use CNN-CRF [13,21] structure to achieve the speed and effectiveness of many NLP tasks.

In recent years, the use of external resources has improved the overall effect of named entity recognition. For example, Peters et al. [22] capture the high-level state of context-related information and the low-level state of modeling syntax through the linear combination of the superimposed internal hidden states in the deep bidirectional language model ELMo [23,24,25]. Sun et al. [26] viewed biomedical entities as machine reading comprehension (MRC) problems and used BERT to perform biomedical named entity recognition in the MRC framework to improve the ability of the model to identify target entities. Guo et al. [27] combined the transformer with the soft item position lattice to form a soft lattice structure transformer and simulated a network similar to LSTM, which is superior to the LSTM network. The overall effect of the model applies to Chinese clinical records. Li et al. [28] proposed the NEAT model to extract named entities. It effectively combines the classic rule-based and dictionary extractors with the context language model to capture special names. The model alleviates the noise problem in advertising text. Alsaaran et al. [29] proposed the BGRU-CRF named entity recognition model based on BERT. The experiment shows that it is superior to other classical models and has achieved good results on the classic Arabic NER dataset.

### 2.2. Nested Named Entities

Nested named entities are common in real life and have attracted a growing number of scholars’ attention in recent years. Scholars have proposed many approaches to nested NER, which can be classified as transform-based approaches and span-based approaches.

The transformation-based method finally transforms the complex sequence nesting problem into an annotated sequence labeling task. Wang and Lu et al. [30] constructed the forest structure based on the Stack-LSTM network in a bottom-up way by mapping nested sentences to a specified forest. Lin et al. [31] proposed a sequence-to-block architecture, which first identifies anchor words, that is, possible headers of all entities, then uses a regular phrase structure to identify the entity boundaries of each anchor word, and finally divides these candidate entities into corresponding entity types. Ju et al. [32] proposed a stacked LSTM-CRF to identify nested named entities, the output of each layer is used as the input of the next layer, and the model uses the information encoded in its corresponding internal entities. Straka et al. [33] proposed Bi-LSTM as an encoder and LSTM as a decoder to realize the sequence-to-sequence sequence labeling model.

Span-based methods identify nested entities by classifying subsequences, or text spans, in a given sentence. Luan et al. [34] proposed a general information extraction framework, DYGIE, in which the state-span graph method enhances the interaction between tasks and allows the model to learn useful information from a wider range of contexts. Zheng et al. [35] proposed a boundary sensing neural network model, which detects the span formed by the starting and ending boundaries of entities, and then divides these spans into corresponding entity types or non-entity types. Sohrab and Miwa [36] regarded nested named entity recognition as a classification problem and used the deep exhaustive model to determine whether the set interval L and the interval below L (L is artificially set data) were entities from top to bottom. Huang et al. [37] designed an extractor to extract entities of specific entity categories using a hybrid selection method and used a discriminator to score. In addition, they used GAT to train the extractor and discriminator to reduce the need for labeling. Shen et al. [38] regarded nested NER as a joint task of boundary regression and span classification, regressed the boundary of each span with a regression to locate the left and right boundaries of the entity, and adjusted the boundary of the span according to the output of the regression. The experiment showed that it achieved good results on ACE and other datasets. Yuan et al. [39] introduced such factors as tags, boundaries, labels, and related spans, using boundaries and tags as queries, and using internal tags and related spans as keys and values for span representation to improve span classification. Wan et al. [40] built an entity-entity diagram and cross-entity diagram globally based on n-gram similarity to integrate information of similar adjacent entities into span representation to enhance span-based methods.

### 2.3. Discussion

Non-nested NER assumes no overlap between entities, so they do not apply to nested NER. The nested NER method based on transformation requires some complex transformation and decoding operations, which may lead to cascading errors and high computational costs. The current span-based nested NER methods lack clear boundary supervision and generate many negative samples for span-based classification. In addition, their computational costs are also very expensive. There are some other problems in nested named entity recognition, including weak fusion effect of word semantic features and insufficient detection of boundary information. This paper proposes a nested NER model based on two-flow feature complementation. The model is different from the current span-based method and proposes a dual flow complementary mechanism to obtain more feature information. Finally, it enters the entity judgment module and the fine-grained partition module to identify nested entities. The model fully captures the dependencies between different features and more semantic information and achieves good results.

## 3. Methodology

### 3.1. Overview Network Architecture

**Figure 1:**
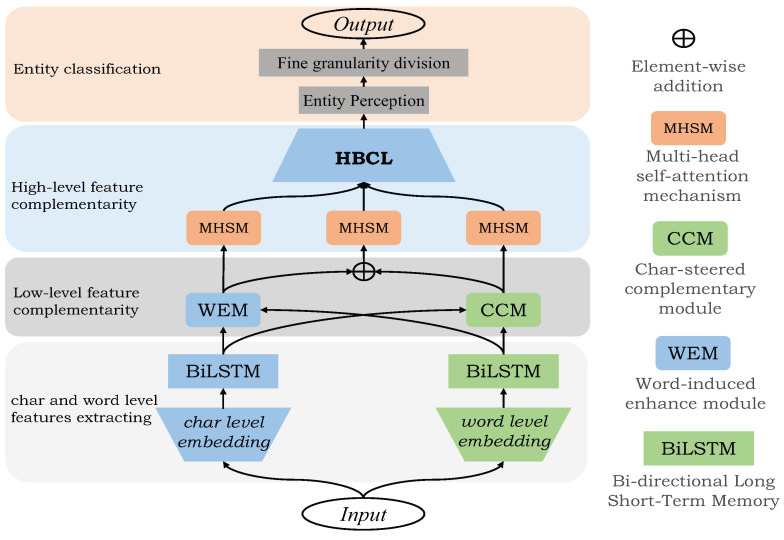
The overall structure of our model. Sending word-level and character-level representation vectors of words into Bi-LSTM to obtain context information of sentences; Then a low-level feature complementary layer is performed, and each feature vector incorporates the effective information of another feature; Then the expression vector of the final sentence is obtained through feature vector fusion in the feature complementary layer of the high-level; Then, the span of entity words is determined to enter the fine-grained division interval to determine whether there are nested entity words, and finally enter the full connection layer and softmax layer to obtain the final entity type classification.

The overall model architecture of this paper is shown in Figure 1. The whole model can be divided into four layers: the first layer is the word embedding and feature extraction part, which sends the word-level and character- level representation vectors of words into Bi-LSTM to obtain the context information of sentences; The second layer is a low-level feature complementary layer, and each feature vector adds effective information of another feature to obtain a new feature representation; The third layer is a high-level feature complementary layer, and the final sentence representation vector is obtained through feature vector fusion; The fourth layer is category judgment. The expression vector of the sentence first enters the entity perception module, and the span of entity words is judged to enter the fine-grained division interval to judge whether there are nested entity words. Finally, it enters the full connection layer and the softmax layer [41] to obtain the final entity type classification. The abbreviations used throughout the paper are present in Table 1.

### 3.2. Character Embedding and Feature Extraction

**Figure 2:**
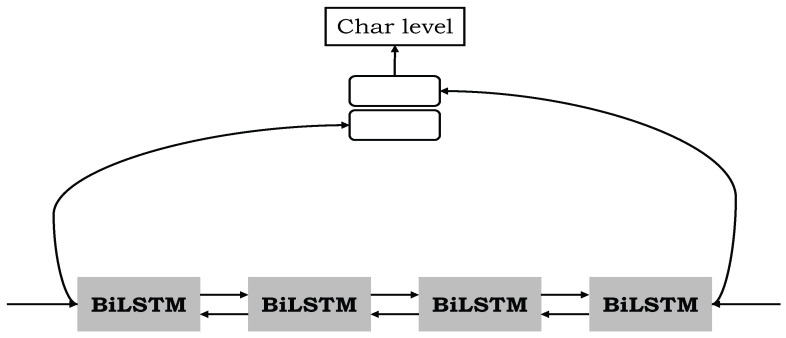
The token representation of a token ‘gene’ in our model. In the process of character-level embedding of words, Bi-LSTM is used to capture the character-level information of words.

Define the input sentence code as s = {s1, s2,…, sn}. There are two kinds of expression vectors embedded in each sentence, which are the word-level expression of words and character-level expression of words. The word-level embedding vector of each word is expressed as $e_{t}^{w}$, and the character-level embedding vector is expressed as $e_{t}^{c}$, where *i* is the ith word of the sentence. The word-level embedding process of words is as follows: First, we build a word vocabulary according to the dataset and then initialize the vocabulary to obtain the word vector table by publicly available pre-training word vectors (We used an open pre-training word vector used in the work of Sohrab and Miwa et al. [36]) or by random initialization. All unknown words outside the word vector table are mapped to the same randomly initialized “UNK” word vector, and the word vector is updated in the continuous training process to obtain the final word representation vector. In the process of character-level embedding of words, Bi-LSTM is used to capture the character-level information of words. First, a character table is constructed for all character, and character vectors are randomly initialized for them. Then, each word is regarded as a determined character sequence. Finally, Bi-LSTM is used to obtain the character-level embedding vector of words. Figure 2 shows the process of obtaining vectors.

In this paper, we define the word-level embedding vector of a sentence as $s^{w}$ and the character-level embedding vector of a sentence as $s^{c}$. Formulas (1) and (2) give two kinds of embedded representation vectors of sentences.

(1) $$\begin{array}{c} s^{w}=\left\{e_{1}^{w},e_{2}^{w},\ldots ,e_{n}^{w}\right\}, \end{array}$$

(2) $$\begin{array}{c} s^{c}=\left\{e_{1}^{c},e_{2}^{c},\ldots ,e_{n}^{c}\right\}, \end{array}$$

where $e^{w}$ represents the pre-trained word vector lookup table, and $e^{c}$ represents the character embedding vector table obtained by Bi-LSTM.

Then, the two embedded vectors are input into Bi-LSTM to obtain the context information of the sentence, and the word-level feature representation vector and the character-level feature representation vector are obtained. Taking the word-level feature representation vector as an example, formulas (3)–(8) calculate the hidden state of each cell at that time.

(3) $$\begin{array}{c} i_{t}^{w}=\sigma \left(W_{i}^{w}\cdot \left[h_{t-1}^{w};e_{t}^{w}\right]+b_{i}^{w}\right) \end{array}$$

(4) $$\begin{array}{c} f_{t}^{w}=\sigma \left(W_{f}^{w}\cdot \left[h_{t-1}^{w};e_{t}^{w}\right]+b_{f}^{w}\right) \end{array}$$

(5) $$\begin{array}{c} u_{t}^{w}=tanh\left(W_{u}^{w}\cdot \left[h_{t-1}^{w};e_{t}^{w}\right]+b_{c}^{w}\right) \end{array}$$

(6) $$\begin{array}{c} o_{t}^{w}=\sigma \left(W_{o}^{w}\cdot \left[h_{t-1}^{w};e_{t}^{w}\right]+b_{o}^{w}\right) \end{array}$$

(7) $$\begin{array}{c} c_{t}^{w}=f_{t}^{w}\cdot c_{t-1}^{w}+i_{t}^{w}\cdot u_{t}^{w} \end{array}$$

(8) $$\begin{array}{c} h_{t}^{w}=o_{t}^{w}\cdot tanh\left(c_{t}^{w}\right) \end{array}$$

where · is element-wise multiplication; [;] is cat operation; $i_{t}^{w}$, $f_{t}^{w}$, $o_{t}^{w}$ denote an input gate, a forgetting gate, and an output gate respectively. The functions are to determine the information to be added to the cell state, what information to discard from the cell state and output the processed value based on the contents saved in the cell state. $u_{t}^{w}$ represents the state candidate value, $c_{t}^{w}$ represents the latest state of the memory cell at that time. $W^{w}$ is the weight matrix output by the unit, and $b^{w}$ represents the offset vector.

To effectively use the context information, a bidirectional LSTM structure is used to input the text forward and backward to obtain two different intermediate layer representations. Finally, the final hidden layer output is obtained by splicing:

(9) $$\begin{array}{c} \vec{h_{i}^{w}}=LSTM\left(\vec{h_{i-1}^{w}},e_{i}^{w}\right) \end{array}$$

(10) $$\begin{array}{c} \overset{\leftarrow }{h_{i}^{w}}=LSTM\left(\overset{\leftarrow }{h_{i-1}^{w}},e_{i}^{w}\right) \end{array}$$

(11) $$\begin{array}{c} h_{i}^{w}=\left[\vec{h_{i}^{w}};\overset{\leftarrow }{h_{i}^{w}}\right] \end{array}$$

where $\vec{h_{i}^{w}}$ and $\overset{\leftarrow }{h_{i}^{w}}$ represent the forward and backward representations of the LSTM at the *i*th position, respectively. Similarly, the character-level feature representation vector at the *i*th position of the sentence can be obtained as $h_{i}^{c}=[\vec{h_{i}^{c}};\overset{\leftarrow }{h_{i}^{c}}$].

### 3.3. Low-Level Feature Complementarity

**Figure 3:**
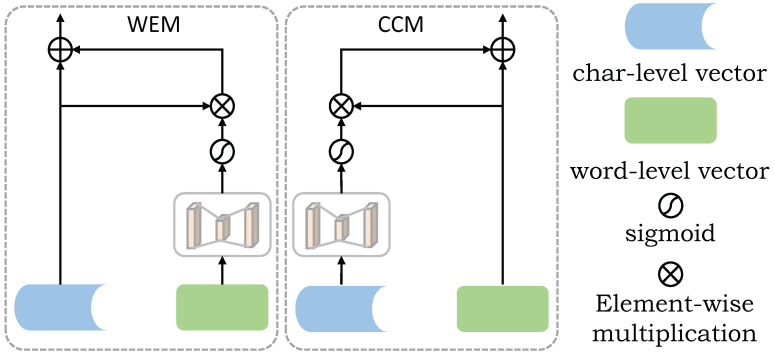
The structures of the WEM and CCM. WEM(CCM) obtains a new word (character)-level embedded feature vector after low-level feature complementation. The character (word)-level embedded feature vector obtains the feature weight through the activation function, and then the weight is multiplied with the character (word)-level embedded feature vector to extract the useful feature of the character (word)-level embedded. Finally, the new word (character)-level embedded feature vector is obtained through residual connection.

This layer belongs to low-level feature complementation, which not only focuses on the feature dependency between different word expression vectors but also will obtain more low-level semantic information. As shown in Figure 3, the process of low-level feature complementation is given.

In Figure 3, CCM refers to the new word-level embedded feature vector after supplement. MLP [42] refers to multi-layer perceptron, which is used to extract low-level semantic information. The character-level embedded feature vector obtains the feature weight through the sigmoid activation function, and then the weight is multiplied with the word-level embedded feature vector to extract the useful features of the character-level embedded. Finally, a new word-level embedded feature vector is obtained through residual connection. Equation (12) gives the operation process.

(12) $$\begin{array}{c} \bar{h_{i}^{w}}=\left[\left(sigmoid\left(MLP\left(h_{i}^{c}\right)\right)\cdot h_{i}^{w}\right);h_{i}^{w}\right] \end{array}$$

Similarly, WEM refers to a new character-level embedded feature vector after feature complementation. The whole operation process is similar to the generation of CCM. First, the low-level semantic information is extracted by the multi-layer perceptron MLP, and the weight is obtained by activating the sigmoid function. Then, the weight is multiplied by the character-level embedded vector to obtain the useful features contained in the character-level embedded vector. Finally, the new character-level embedded feature vector is obtained by residual connection. Formula (13) gives the whole operation process.

(13) $$\begin{array}{c} \bar{h_{i}^{w}}=\left[\left(sigmoid\left(MLP\left(h_{i}^{w}\right)\right)\cdot h_{i}^{c}\right);h_{i}^{c}\right] \end{array}$$

### 3.4. High-Level Feature Complementarity

#### 3.4.1. Multi-Head Self-Attention Mechanism

This paper adds a multi-head attention mechanism [43] to this module, which makes the model focus more on important features, reduces the attention to non-important features, and optimizes resource allocation. MHSM in the overall model framework of this paper is the multi-head attention mechanism.

We need to calculate the weight of attention, firstly, enter *Q* (query) and *K* (key) of dimension, *V* (value) of the dimension, and then calculate the similarity of *Q* and *K* to obtain the weight. After the dot product of *Q* and *K*, divide by $\sqrt{d_{k}}$ (to prevent the gradient from disappearing), and use a softmax function to obtain a weight of *V*. The calculation process is shown in formula (14).

(14) $$\begin{array}{c} Attention\left(Q,K,V\right)=softmax\frac{QK^{T}}{\sqrt{d_{k}}}V \end{array}$$

Multi-head self-attention is used to connect multiple self-attention, which can be formatted as:

(15) $$\begin{array}{c} MultiHead\left(Q,K,V\right)=Concat\left(head_{1},\ldots ,heaad_{h}\right)W^{o} \end{array}$$

(16) $$\begin{array}{c} head_{i}=Attention\left(QW_{i}^{Q},KW_{i}^{K},VW_{i}^{V}\right) \end{array}$$

where *K*, *Q*, *V*, $W^{o}$ are the trained weight parameters, and *h* is the number of attention heads. $head_{i}$ is a single head attention unit, which is set to 8. $W_{i}^{Q}$, $W_{i}^{K}$, $W_{i}^{V}$ represent a trainable weight matrix.

#### 3.4.2. HBCL

**Figure 4:**
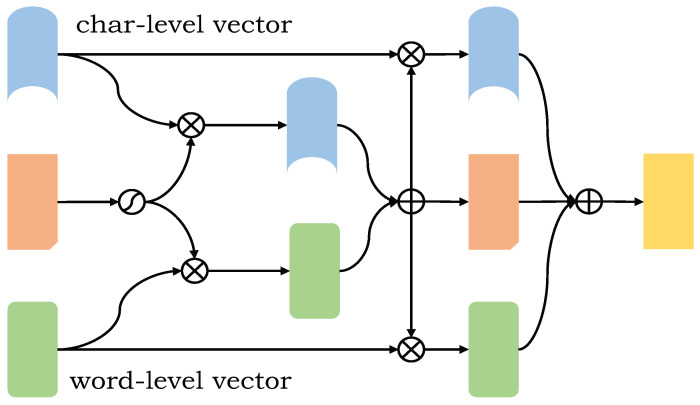
The structure of the HBCL. When high-level feature complementation generates a new word-level embedded feature vector and character-level embedded feature vector, feature complementation is performed twice, which greatly improves the acquisition of effective features and reduces the proportion of useless features.

After the local features are obtained by the multi-head attention mechanism, the two feature vectors can be further complemented to obtain deeper semantic information. The features of the whole high level are complementary as shown in Figure 4.

A fused vector is the fusion of word-level embedded feature vector and a character-level embedded feature vector. It is connected by vectors and recorded as $\bar{h_{i}}\left(\bar{h_{i}}=\left[\bar{h_{i}^{w}}:\bar{h_{i}^{c}}\right]\right)$. Then, the weight H of the feature is obtained through the activation function. The word-level feature vector and the character-level feature vector are respectively multiplied by the weight to obtain a new feature vector. At this time, high-level feature complementation is carried out. The whole process is shown in formulas (17)–(19).

(17) $$\begin{array}{c} H=sigmoid(\bar{h_{i}}) \end{array}$$

(18) $$\begin{array}{c} \hat{h_{i}^{w}}=H\cdot \bar{h_{i}^{w}} \end{array}$$

(19) $$\begin{array}{c} \hat{h_{i}^{c}}=H\cdot \bar{h_{i}^{c}} \end{array}$$

$\hat{h_{i}^{w}}$ and $\hat{h_{i}^{c}}$ after activation, the weight matrices $H^{w}$ and $H^{c}$ are obtained, which are respectively multiplied with the two input eigenvectors $\bar{h_{i}^{w}}$ and $\bar{h_{i}^{c}}$ to obtain a new eigenvector $\tilde{h_{i}^{w}}$ and $\tilde{h_{i}^{c}}$, finally, the final expression vector at the sentence level is obtained. The whole process is shown in formulas (20)–(24).

(20) $$\begin{array}{c} H^{w}=sigmoid\left(\hat{h_{i}^{w}}\right) \end{array}$$

(21) $$\begin{array}{c} H^{c}=sigmoid\left(\hat{h_{i}^{c}}\right) \end{array}$$

(22) $$\begin{array}{c} \tilde{h_{i}^{w}}=H^{w}\cdot \hat{h_{i}^{w}} \end{array}$$

(23) $$\begin{array}{c} \tilde{h_{i}^{c}}=H^{w}\cdot \hat{h_{i}^{c}} \end{array}$$

(24) $$\begin{array}{c} H_{i}=\left[\tilde{h_{i}^{w}};\bar{h_{i}};\tilde{h_{i}^{c}}\right] \end{array}$$

High-level feature complementation generates a new word-level embedded feature vector $\tilde{h_{i}^{w}}$ and character-level embedded eigenvector $\tilde{h_{i}^{c}}$. It is necessary to change the feature weight twice, that is, to complement the features twice, which greatly improves the acquisition of effective features and reduces the proportion of useless features.

### 3.5. Entity Classification

After feature complementation, the final expression vector of the sentence first enters the entity word judgment module, and each text is marked with a binary sequence marking method. The entity word is marked as 1 and the non-entity word is marked as 0. This can filter some non-physical areas. Specifically, the feature sequence passes through a full connection layer and sigmoid activation function to obtain the entity word probability *p* (when *p* is greater than 0.5, it is regarded as an entity word) that each word belongs to the internal or boundary of the entity.

The entire span is the span interval of entity words and then enters the fine-grained division module to identify internal entities. For each entity word span interval (the number of words is greater than or equal to 3) $span_{\left(i,j\right)}=\left\{s_{i},s_{i+1},\ldots ,s_{j}\right\}$, which defines the sentence-level feature vector of the starting word is used to represent the left boundary information, and the sentence-level feature vector $H_{j}$ of the last word is used to represent the right boundary information, and the information in the middle part is the average value of the sentence-level feature vectors of all words in the interval. The splicing of the three forms the final representation of the span, that is, $S_{\left(i,j\right)}=\left\{H_{i},\frac{1}{j-i+1}\sum_{k=i}^{j}H_{k},H_{j}\right\}$, where $H_{k}$ is the sentence-level feature representation vector of the $k^{th}$ word.

**Figure 5:**
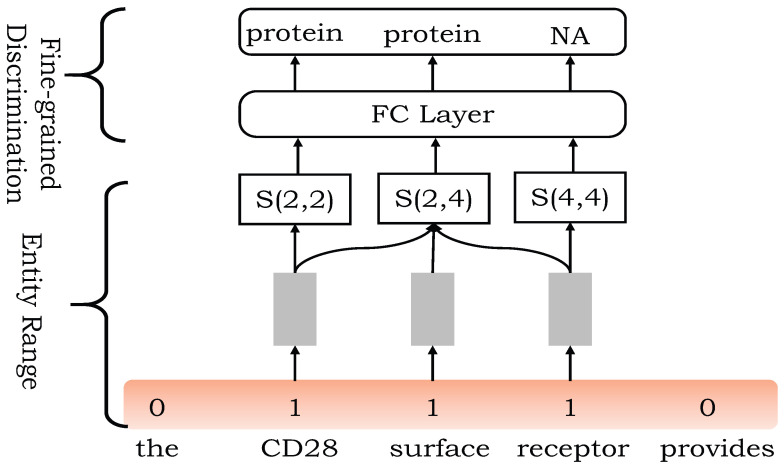
The span interval with the whole span of entity words enters the fine-grained division module to identify internal entities, and fine-grained division can be used to identify internal nested entities. The divided interval enters a full connection layer and softmax layer to get the final category mark.

The fine-grained division of entity word span can be used to identify nested entities. The finely divided interval enters a full connection layer and softmax layer to get the final category mark. The whole process is shown in Figure 5.

In the training process, the cross entropy loss [44] is used as the model structure loss function. The loss function in this paper is composed of two parts. One part is the loss function formed by judging whether the text is an entity word, which is recorded as $L_{l}$. As shown in formula (25):

(25) $$\begin{array}{c} L_{l}=-\left[ylog\left(p\right)+\left(1-y\right)log\left(1-p\right)\right] \end{array}$$

where *y* refers to the real label (1 refers to entity word, 0 refers to non-entity word) that discriminates the element as an entity word, and *p* refers to the probability that discriminates the element as an entity word.

Another part of the loss function, which is recorded as $L_{h}$, is whether the classification is correct when a certain entity word interval < I, J > is finely divided. The cross-entropy loss function is adopted, as shown in formula (26):

(26) $$\begin{array}{c} L_{h}=-\sum_{s=i}^{j}\sum_{c=1}^{N}y_{h,c}log\left(P_{h,c}\right) \end{array}$$

where $y_{h,c}$ indicates whether entity words belong to the binary label of entity category *c* (1 indicates belonging, 0 indicates not belonging), $P_{h,c}$ indicates the probability that entity words belong to entity category *c*, and there are *N* entity categories in total.

Therefore, the loss *L* of the model in this paper on the training set is the weighted average of the multi-task training loss. As shown in formula (27):

(27) $$\begin{array}{c} L=\lambda \sum_{i=1}^{x}L_{l}\left(s_{i}\right)+\left(1-\lambda \right)L_{h}\left(span_{i}\right) \end{array}$$

where $s_{i}$ of the first item represents the *i*th text and *x* represents the number of text, $span_{i}$ of the second item represents the *i*th entity word span, and *t* represents the number of entity word spans. λ is a super parameter (0 < λ < 1), and it represents the weight of judging whether the element is an entity word in the whole model loss.

## 4. Experiments

### 4.1. DataSet and Annotation Method

To verify the validity of the model, the dataset used in this paper is the preprocessed version published by GENIA [45]. The GENIA dataset contains five entity categories, namely DNA, RNA, Protein, cell line, and cell type. The training set, development set, and test set of this paper will be tested according to the ratio of 8.1:0.9:1. Table 2 gives the statistical data of this dataset.

Different datasets may use different annotation methods. The common annotation methods are the BIOES annotation method, Markup annotation method, and BIO annotation method. The dataset selected in this paper is the BIO annotation method. “B-X” indicates that the fragment of this element belongs to type X and this element is at the beginning of this fragment, “I-X” indicates that the fragment of this element belongs to type X and this element is in the middle of the fragment, “O” indicates that it does not belong to any type.

Before labeling, the maximum number of nesting layers N (N is set to 4) shall be obtained. During labeling, N columns shall be marked for each word, and category information shall be marked from the inner layer to the outer layer. Table 3 shows the nested entity annotation results of a segment in the GENIA dataset.

### 4.2. Experimental Parameters and Environment

The model in this paper is based on PyTorch 1.3.1 framework. The pre-trained word-level embedded vector dimension and character-level embedded vector dimension are each 200 dimensions and are initialized randomly. The model parameters are shown in Table 4. The specific experimental environment settings are shown in Table 5.

### 4.3. Evaluation Criterion

In the experiment, the precision rate (P), recall rate (R), and comprehensive evaluation index F1 were used as the evaluation indexes of the model performance. The accuracy rate refers to the probability that the elements predicted by the model are entities, and the recall rate refers to the probability that all entities in the dataset are accurately identified by the model.

### 4.4. Comparison of Experimental Results

To verify the effectiveness of two kinds of embedded eigenvectors for dual stream complementation in this paper, the following five representative models are selected as the baseline model, and the selectional results are as follows:

1. Lu and Roth [46] jointly modeled and identified entity boundaries, entity types, and entity heads based on the Hypergraph method for the first time.
2. Xu and Jiang et al. [47] proposed the method based on local detection, which is superior to the traditional sequence labeling method without any external resources or feature engineering;
3. Sohrab and Miwa [36] listed all possible interval spans as potential entity segments, and then used a deep neural network to classify them;
4. Ju and Miwa et al. [32] proposed a new neural model to identify nested entities by dynamically stacking non-nested NER layers, and the cascaded CRF layer was used to extract information encoded by internal entities in an internal to external manner to identify external entities;
5. Lin and Shao et al. [48] proposed combining the hypergraph model with a neural network to identify overlapping elements by hypergraph and recognize nested entities by neural network acquiring features;

Table 6 shows the experimental results of different models on the GENIA dataset.

From the experimental results, the R-value and F1-value in this paper are optimal. Among them, the model proposed by Ju and Miwa may be better because CRF can learn the constraint conditions from the training data, thus improving the effectiveness of the prediction tag, but the model may generate cascading errors. Lin and Shao et al. use the hypergraph model to identify nested results. This is not as effective as other models, which may be due to the large gap between the mapping between the actual nested structure and the graph structure. The experiment shows that the model in this paper is superior to the former. Specifically, the R-value of this model is 8.3% higher than that of Lin and Shao, and the F1-value is 1% higher than that of Ju and Miwa.

### 4.5. Ablation Studies

Feature extraction is an important step and key part of nested named entity recognition, so it is very important to consider the extraction of feature information when designing the model. To verify the effect of this model on feature recognition based on dual stream feature complementation, the following five models are designed for comparison, and experiments and analysis are carried out on the GENIA dataset. All models adopt two embedding methods, word-level embedding, and character-level embedding.

1. Bi-LSTM + MHSM: the two embedded vectors use Bi-LSTM to extract context feature information, and then use a multi-head attention mechanism to obtain local feature information.
2. Bi-LSTM + CCM + MHSM + HBCL: after using Bi-LSTM to obtain the text context information features, the effective information of the character-level embedded feature vector is added to the word-level embedded feature vector, and then the multi-head attention mechanism is entered, and high-level feature complementation is performed.
3. Bi-LSTM + WEM + MHSM + HBCL: after obtaining two kinds of feature vectors through Bi-LSTM, the effective information of the word-level embedded feature vector is added to the character-level embedded feature vector, and then local feature information is obtained, and then high-level feature complementation is performed.
4. Bi-LSTM + MHSM + HBCL: use Bi-LSTM to obtain the text context feature information, then obtain the sentence local feature information, and then directly carry out high-level feature complementation to obtain the sentence feature vector.
5. Bi-LSTM + CCM + WEM + MHSM: after obtaining the two feature vectors through Bi-LSTM, the low-level feature complementation of the two dimensions is carried out, and then the multi-head attention mechanism is entered to obtain the local feature information of the sentence.

The results of each model of the ablation experiment are shown in Table 7.

Comparing the model in this paper with the ablation model (1), it can be seen that the extraction performance of feature information is significantly improved after the two flow feature complementation, in which the R-value is increased by 9.1%, and the F1-value is increased by 4.1%. Comparing the ablation model (1) with the ablation models (2) and (3), it can be seen that the extraction effect of feature information is improved after adding feature complementation of a certain dimension. This is because low-level semantic information can be obtained after low-level feature complementation, thus improving the overall recognition effect. From the comparison of the results of the ablation model (1) and ablation model (4), it can be found that high-level feature information complementation is also very important for feature information extraction. Comparing the model in this paper with the ablation model (5), we can find the importance of high-level feature complementation. After the low-level feature complementation, the high-level feature complementation is added, in which the R-value is increased by 5.6%, and the F1-value is increased by 1.6%. Comparing ablation models (4) and (5), it can be found that the F1-value of only low-level feature complementation is slightly higher than that of only high-level feature complementation, which may be because the high-level feature complementation depends on the low-level feature complementation to a certain extent.

Based on the above experimental results, it can be concluded that the feature vectors obtained by the two different embedding methods enhance the extraction of useful feature information after integrating the low-level feature complementation and the high-level feature complementation, thus improving the overall recognition effect of nested named entities.

## 5. Conclusions

In this paper, we propose a model based on two-stream feature complementation to obtain efficient and useful feature information. In this model, a multi-head attention mechanism is used to mine the local information of sentences. Meanwhile, this paper uses word-level embedding and character-level embedding to obtain the features of sentences. The results show that the sentence representation vectors obtained from the two dimensions improve the recognition effect of nested entities after the feature complementation operation. It is proved that the method in this paper captures the hidden low-level semantic information and deep semantic information, and provides more favorable feature information for subsequent entity classification.

Some studies have found that the latest pre-training language models such as Roberta or ALBERT can obtain good initialization effects, handle the prediction within sentences, and capture the dependency between words across sentences. It can be seen that if the transformer-based pre-training model is used in the sentence embedding process, it will bring great advantages. Therefore, in future research work, we will combine some pre-training language models to obtain sentence feature information, and we will continue to study how to efficiently decode tags in nested named entities.

## Figures and Tables

**Table 1:** Abbreviations of terms.

Terms	Abbreviations	Function
WEM	word-induced enhance module	This module obtains word-level embedded feature vectors after low-level feature complementation.
CCM	char-steered complementary module	This module obtains character-level embedded feature vectors after low-level feature complementation
MHSM	multi-head self-attention mechanism	It makes the model pay more attention to important features, reduces the attention to non important features, and optimizes resource allocation.
HBCL	Height bi-directional interactive connect layer	Height bi-directional interactive connect layer Two word representation vectors complement high-level features to obtain the final sentence representation vector.

**Table 2:** GENIA dataset.

Item	Train	Dev.	Test	Overall	Nested
DNA	7650	1026	1257	9933	1774
RNA	692	132	109	933	407
Protein	28,728	2303	3066	34,097	1902
Cell line	3027	325	438	3790	347
Cell type	5832	551	604	6987	389
Overall	45,929	4337	5474	55,740	4789

**Table 3:** BIO annotation fragment.

Element	First-Level Annotation	Second-Level Annotation	Third-Layer Annotation	Fourth-Layer Annotation
IL	B-protein	B-RNA	O	O
-	I-protein	I-RNA	O	O
2R	I-protein	I-RNA	O	O
alpha	I-protein	I-RNA	O	O
mRNA	O	I-RNA	O	O
that	O	O	O	O
is	O	O	O	O

**Table 4:** Experimental parameter setting.

Parameter Type	Parameter Value
dropout loss rate	0.5
batch size	50
word-level embedded vector dimension	200
character-level embedded vector dimension	200
LSTM hidden layers	200
LSTM layers	1
number of attention mechanism heads	8
learning rate	0.0005
Epoch	60

**Table 5:** Experimental environment settings.

Object	Environment
system	window 10
GPU	NVIDIA GTX 2080Ti GPU
hard disk	200G
memory	64G
Python version	Python 3.7
Pytorch	1.3.1

**Table 6:** Experimental results of different models on GENIA.

Model	P	R	F1
Lu & Roth	72.5	65.2	68.7
Xu & Jiang	71.2	64.3	67.6
Sohrab & Miwa	73.3	68.3	70.7
Ju & Miwa	76.1	66.8	71.7
Lin & Shao	70.3	68.9	69.6
Ours	68.7	77.2	72.7

**Table 7:** Results of ablation studies on GENIA.

Model	P	R	F1
Bi-LSTM + MHSM	69.1	68.1	68.6
Bi-LSTM + CCM + MHSM + HBCL	67.5	75.9	71.5
Bi-LSTM + WEM + MHSM + HBCL	66.0	77.7	71.4
Bi-LSTM + MHSM + HBCL	66.1	72.6	69.2
Bi-LSTM + CCM + WEM + MHSM	70.7	71.6	71.1
Ours	68.7	77.2	72.7

## Data Availability

Not applicable.
